# Improving dependability with low power fault detection model for skinny-hash

**DOI:** 10.1371/journal.pone.0316012

**Published:** 2024-12-19

**Authors:** Sonal Arvind Barge, Gerardine Immaculate Mary

**Affiliations:** School of Electronics Engineering, Vellore Institute of Technology, Vellore, Tamil Nadu, India; State University of New York at Oswego, UNITED STATES OF AMERICA

## Abstract

The increasing popularity and prevalence of Internet of Things (IoT) applications have led to the widespread use of IoT devices. These devices gather information from their environment and send it across a network. IoT devices are unreliable due to their susceptibility to defect that arise intentionally or spontaneously. IoT devices must be dependable and secure since they form an integral part of the network that connects millions of connected objects. IoT devices are secured by cryptographic algorithms, but their dependability is a major concern. Concurrent error detection (CED) techniques, sometimes referred to as fault detection techniques, are extensively employed to enhance the dependability of embedded devices. Two fault detection approaches are proposed to detect faults in cryptographic algorithms running on IoT devices. Recomputing with complemented operands (RECO) and Double modular redundancy with complemented operands (DMRC) is proposed. Generally, IoT applications deploy resource-constrained devices and cannot support high-level security techniques. Therefore, the mentioned fault detection technique is assimilated for the lightweight SKINNY block cipher. The resource-sharing concept is applied to the SKINNY block cipher to reduce area overhead caused by DMRC. The SKINNY-Hash function construct is described using Very Large-Scale Hardware Description Language (VHDL). Functional behaviour is tested using ModelSim SE-64. The proposed architecture is synthesised using the Genus synthesis tool by Cadence, and area-power reports are generated. The proposed work is compared with the other CED techniques in terms of area and power consumption, and the work proves to have less overhead.

## Introduction

Embedded devices’ dependability is adversely affected by technology scaling [1], which makes them more vulnerable to naturally occurring and forcefully injected faults. Faults are categorised into two types: transient and permanent faults. These faults occur due to supply voltage variation, short connections, open connections, etc. or external causes such as electromagnetic radiation [2, 3]. These faults impair the functionality of combinational and sequential circuits in integrated circuits of embedded devices. IoT device vulnerabilities can be exploited by attackers in several ways. The fault attacks interfere with the normal operation of the integrated circuit of the embedded device. The attacker uses these disruptions to target cryptographic algorithms running on the embedded device [4]. The IoT devices are easy targets for attackers stationed in easily accessible locations. Most IoT applications deploy resource-constrained devices, and incorporating conventional cryptographic algorithms is unsuitable as it requires more memory, area, and power. Lightweight cryptography is a favourable solution for resource-constrained devices such as IoT, offering high security, less hardware complexity, and low power consumption [5]. Even so, cryptographic algorithms are prone to fault attacks to acquire the secret key [6]. Several CED techniques have been studied to detect faults in hardware architectures. CED techniques observe the operational behaviour of the system to detect any deviation from normal operation functionality. These techniques are categorised as hardware (space), time, data, and software redundancy [7]. Fault detection offers high system dependability but has a negative impact on embedded systems as well, such as time overhead, more power consumption, and high temperature.

Space or hardware redundancy duplicates the hardware modules to verify functionality by comparing the output of respective modules. This technique has 100% area overhead and impacts the weight, power consumption, and cost of the system [8]. Detecting faults is also quite complicated if all the hardware modules in hardware redundancy are faulty. Time redundancy is also called computation redundancy, where computation is repeated, and the functionality of the module is by comparing the results with previously stored computation outputs [9]. A mismatch in results indicates a fault existence in the hardware module. This technique detects both transient and permanent faults but still has a drawback of throughput overhead [10]. On the other hand, data redundancy, also known as information redundancy, generates check bits from input message to propagate with input message bits; these check bits verify faults in the data while being transmitted or stored in memory [11]. Researchers have also studied mixed redundancy, known as hybrid redundancy [12], to achieve a balance between reliability, area, and power consumption.

In this article, SKINNY-Hash is adapted for the application of the proposed fault detection technique. Skinny-hash is a lightweight cryptographic hash function designed for constrained environments, such as IoT devices. It has a small footprint, requiring minimal computational resources, which is essential for devices with limited processing power. Skinny-hash is optimized for speed, enabling quick data processing, which is crucial for real-time applications in IoT. It can be applied across various IoT scenarios, from smart home devices to industrial applications, providing a consistent hashing solution. Being a standardized hashing method allows for easier integration with different platforms and systems, facilitating communication among diverse IoT devices. The straightforward design of SKINNY-Hash can make implementation easier, reducing the complexity often associated with cryptographic functions. SKINNY- hashing algorithms often prioritize efficiency and speed, which can make them vulnerable to certain types of failures or attacks. Fault tolerance helps prevent vulnerabilities that could be exploited by attackers. If a hash function can be manipulated due to a fault, it could lead to collisions or predictable outputs.

To counter fault injection attacks, two main categories of countermeasures are proposed: detection and infection. Detection countermeasures identify faults during hardware execution. When a fault is detected, the hardware avoids outputting any faulty ciphertexts, thereby preventing potential exploitation. Infection countermeasures aim to prevent the misuse of faulty ciphertexts by altering the logical outcome of a fault, significantly changing the results. This makes it difficult for attackers to exploit the erroneous ciphertexts. The National Institute of Standards and Technology (NIST) provided the guidelines about security requirements for cryptographic modules in FIPS PUB 140–2 [13]. It outlines four security tiers. The fourth level highlights that, in order to identify and respond to any unwanted attempts at physical access, the physical security systems must completely safeguard the cryptographic module at the highest level of security. Therefore, this study mainly concentrates on detection.

### Related work

Previous studies have investigated error detection strategies on a range of symmetric-key and public cryptosystems [14–17]. Some researchers apply combined fault detection schemes to improve fault coverage as much as possible in a cryptographic algorithm. Recomputing with negating and swapping is proposed for the Number Theoretic Transform multiplication technique used in post-quantum cryptography [18]. A less complex time redundancy technique named recomputing with rotated operands (RERO) for the secure hash algorithm (SHA) [19]. RERO offers low hardware overhead and is hence suitable for resource-constraint applications. Secure hash architectures are widely used in various applications [20]. Totally Self-Checking (TCS) hashing core is proposed in [21]. It incorporates data and space redundancy together with a TSC checking circuit for SHA-1 and SHA-256. Hardware redundant TSC block and data redundant TSC block are connected by two rail checker (TRC) blocks that generate parity during normal operation and serve parity inputs for the second component. Here, TRC performs data verification and parity generation of output simultaneously, hence less hardware overhead. However, the proposed work provides 100% fault detection for odd faulty bits. Fault coverage for even faulty bits is not considered.

Time redundancy-based CED technique, recomputing with permuted operands, is applied to advance encryption standard (AES) and AES-inspired hash Grøstl. It is observed that a lower fault miss rate is achieved at the expense of a high area-power overhead [22]. A low area-power overhead solution is presented [23], where rounds of AES are divided into three parts with two pipeline registers. Each round output is stored in the respective pipeline register before the re-encryption process to detect faults. This approach improves the frequency of AES round operations. Though it offers better fault coverage, the permanent fault present in the round operations can not be detected. A double parity bit-based data redundancy is proposed and applied to two different AES SBoxes schemes, focusing on transient faults [24]. The author proposes input parity prediction for the first scheme and compares predicted parity with actual parity to detect a fault. For the second scheme, the presence of a transient fault causes the output of the shift-row operation to produce the opposite value.

Some factors, such as the state bit count of block cipher, the size of control logic, and the function unit in a round function, play an important role in designing a hash function with lower area-power overhead [25]. Therefore, parity-bit-based fault detection for lightweight cryptographic algorithms is preferred by some due to minimal hardware overhead [26, 27]. Four methodologies are proposed as signature generators using Hamming codes and parity bits to detect injected faults in AES [28]. The solution provided partially protects the T-boxes and offers complete protection to the rest of the operations involved in the AES block cipher. These signatures can only be deployed to block cipher with memory-based implementation. Three applicable error-correcting 4-bit S-boxes are presented to be used in PRESENT, PRINCE, and lightweight hash function SPONGENT [29]. The error detection and correction operations are performed concurrently with less area and delay overhead. Though fault coverage is 96%, it only detects and corrects faults in S-box.

Hardware redundancy and time redundancy offer maximum reliability for embedded systems that perform arithmetic computation. Hardware or time redundancy combined with encoding schemes can enhance fault detection coverage at the cost of power and area overhead [30]. Thus, two hybrid redundancy-based fault detection methods are deployed in the proposed work for ciphertext computation to achieve maximum fault coverage. Further, the resource-sharing concept is applied to DMRC-SKINNY to achieve less area overhead. Recomputing with complement operands-based fault detection (RECO) and double modular redundancy with complemented operands (DMRC) for lightweight SKINNY-block cipher to improve reliability are proposed. The concepts of mixed redundancy are tailored together to achieve the proposed work.

The contribution and implementation details are as mentioned here:

- A low-power fault-detection technique named RECO and DMRC are proposed for the SKINNY hash function. The self-dual function property is used to detect transient and permanent faults. RECO follows the traditional time redundancy technique with permuted input operands, whereas DMRC follows the hardware redundancy technique with permuted input operands.
- The proposed RECO and DMRC are implemented using VHDL, further synthesised in a 90nm CMOS process technology using Cadence’s Genus design compiler, and functionality is verified using ModelSim SE 2019.4. Transient faults are injected into the architecture through the testbench using linear feedback shift registers (LFSR). Logic lines are forced to either “0” or “1” for permanent fault detection.
- The fault-detection ability against transient and stuck-at-fault is tested. Single-bit, single-byte and multiple-bit faults were injected, and the achieved fault coverage was very high.
- The resource utilisation results are compared with different fault-detection techniques, and it is observed that both the proposed architectures have less area overhead compared to fault-detection techniques in the literature.

### Preliminaries

**Fig 1:**
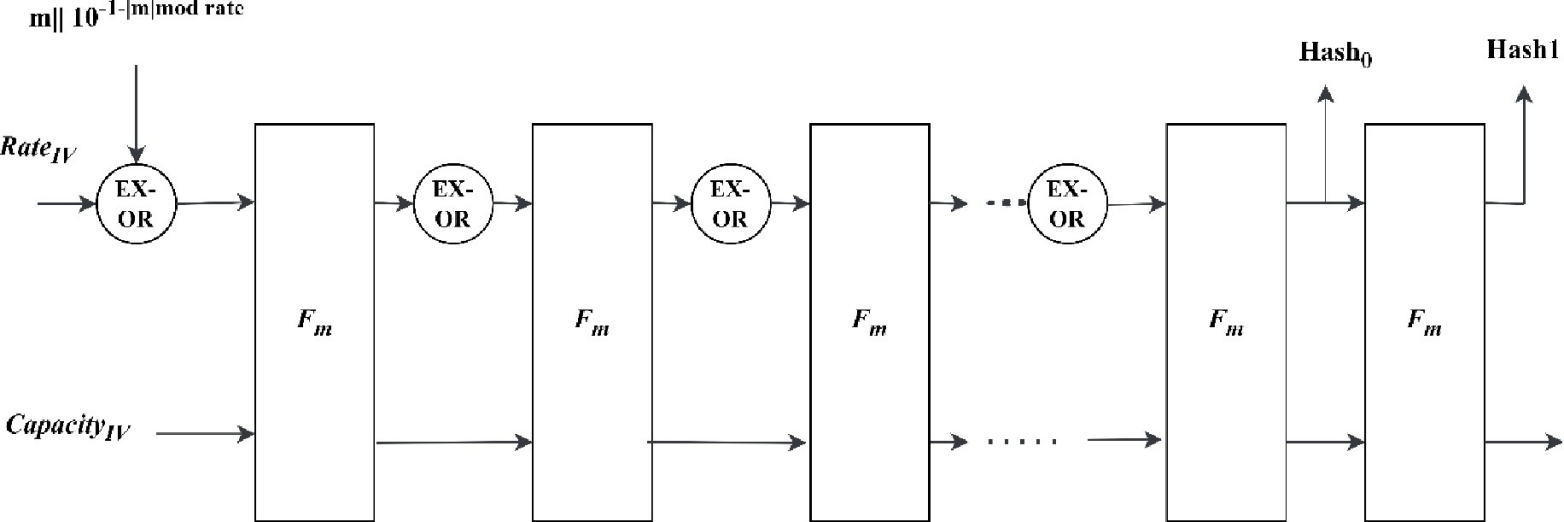
Sponge construct of SKINNY-Hash.

SKINNY-Hash functionality uses SKINNY as a base primitive that belongs to the lightweight tweakable block cipher family [31]. SKINNY-Hash uses sponge construction, which takes the initial vector state and message as input to compute a hash digest. The state is divided into rate and capacity based on the hash functionality chosen for cipher text generation. The message bits are padded with pad10* even though the input message is a multiple of the rate value. During the absorbing phase, the message bits are XORed with rate and given to the function block. 128 bits are extracted during the squeezing phase as the first 128 bits of 256-bit hash. Once again, the state is applied to the hash function to extract the next rate as the last 128-bit of hash. Fig 1 shows the sponge construct where hash function blocks are deployed. These hash functions consist of either primary or secondary members that generate ciphertext. The primary member uses 256-bit tweakey, which is denoted as the SKINNY-tk2 hash function. The secondary member uses 384-bit tweakey and is denoted as SKINNY-tk3 hash functions. The primary member deploys the SKINNY-128-256 block cipher, while the secondary member deploys the SKINNY-128-384 block cipher. The block ciphers receive blocks of the same size, i.e., 128-bit expressed as a square array or vector of cells where each cell is 8-bit. The tweakey size and number of encryption rounds of SKINNY block cipher depend on the member that is used in the hash function.

The proposed work considers both members for fault detection, but here, the hash digest produced using the hash function with the secondary member is described. The internal state vector, *State*_384_ is 384-bit. It is divided into rate *n* and capacity *Capacity*_*IV*_, as shown in (1) and (2)

$$Rate_{IV}=0^{128}$$ (1)

$$Capacity_{IV}=10^{255}$$ (2)

$$State_{384}=Rate_{IV}∥Capacity_{IV}$$ (3)

**Fig 2:**
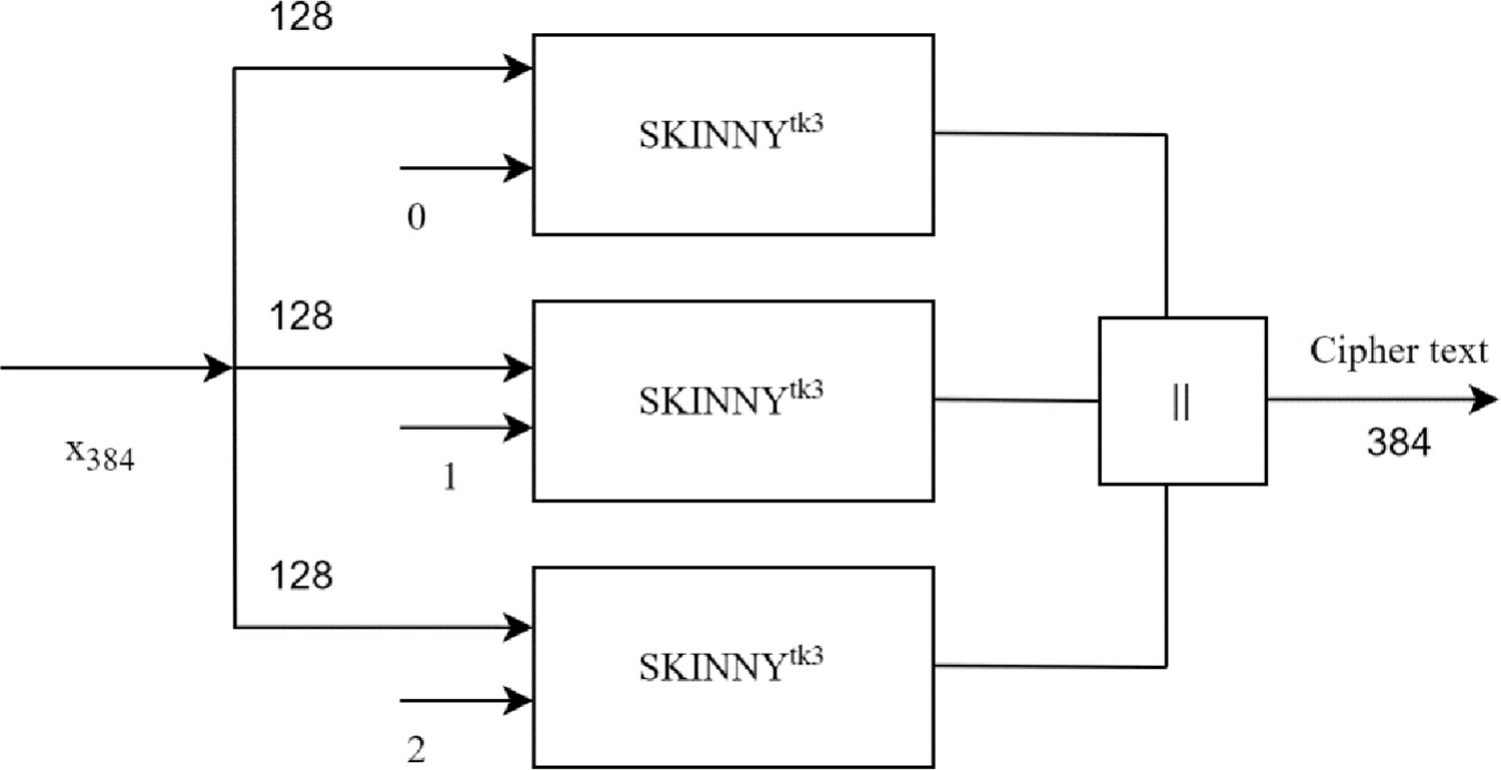
Hash function construct used in SKINNY-tk3-hash.

The construct of a hash function is shown in Fig 2, each block denoted by is a SKINNY-128-384 block cipher used to generate cipher text. The hash function denoted as SKINNY-tk3-Hash receives 384-bit input and produces 384-bit ciphertext.

**Fig 3:**
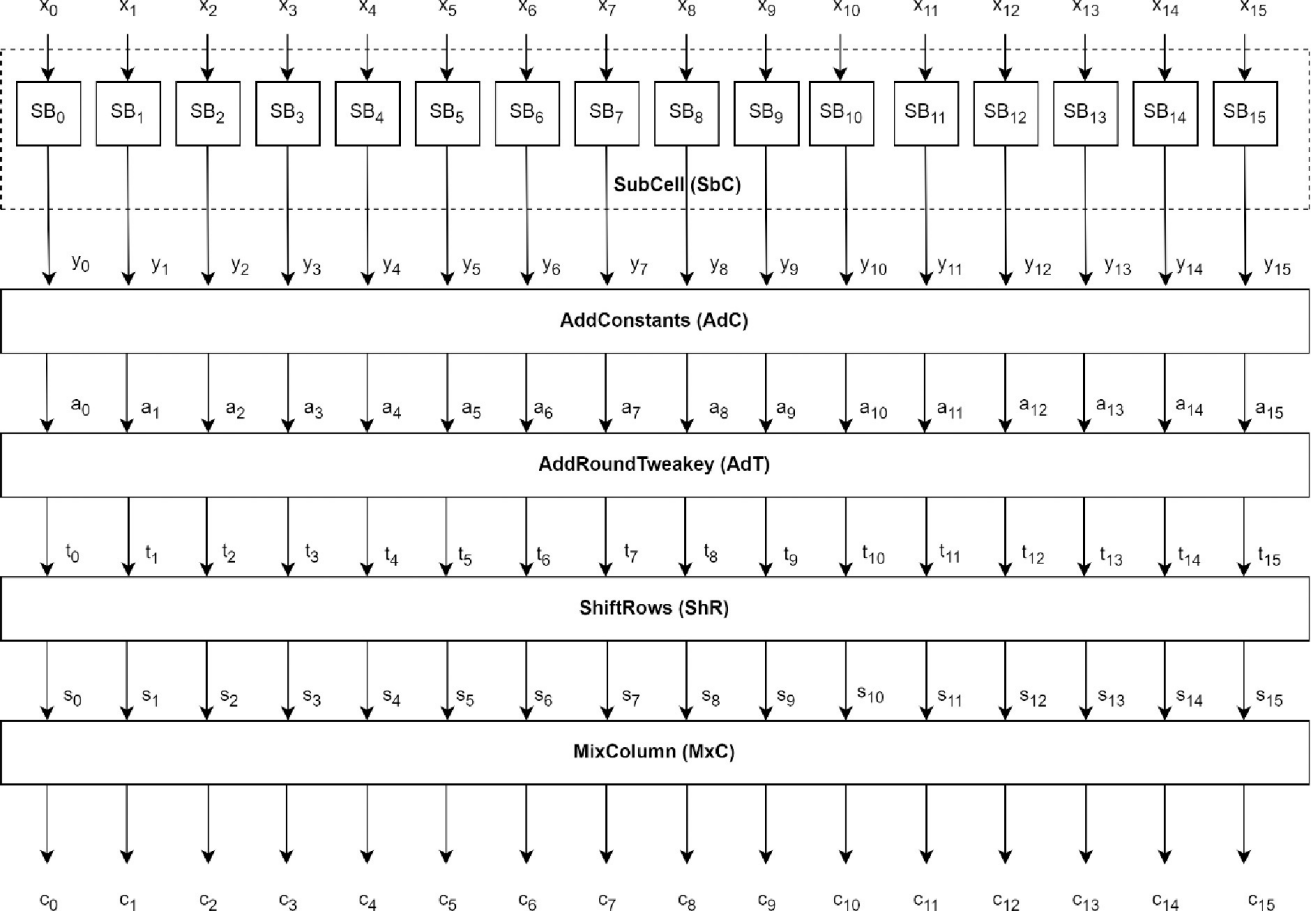
One SKINNY encryption round.

Each SKINNY cipher takes a block of size 128-bit denoted as ‘*n*‘ and tweakey of size denoted as tk1, tk2, and tk3. The SKINNY algorithm follows “TWEAKEY” framework, hence takes tweakey input instead of a separate key and tweak. Fig 3 shows one SKINNY encryption round comprising five operations: *SubCell*, *AddConstants*, *AddRoundTweakey*, *ShiftRows*, and *MixColumn*.

The *Subcell* performs substitution and permutation operations on each 8-bit cell in the internal state. Let *Q* be the internal state array, and an 8-bit *x*_*p*_ is expressed as $x_{p}=i_{7}∥i_{6}∥i_{5}∥i_{4}∥i_{3}∥i_{2}∥i_{1}∥i_{0}$ where *p* = 0,1,2,…15. *i*_*n*_ express the number of bits in each cell where *i*_0_ is the least significant bit, and *i*_7_ is the most significant bit in each cell.


$$Q=[\begin{array}{cccc} x_{0} & x_{1} & x_{2} & x_{3}\\ x_{4} & x_{5} & x_{6} & x_{7}\\ x_{8} & x_{9} & x_{10} & x_{11}\\ x_{12} & x_{13} & x_{14} & x_{15} \end{array}]$$

The *SubCell* operation is performed with a combinational circuit, where each bit of the output cell is derived from the following equations.


$$A=i_{0}⊕\bar{\left(i_{2}\vee i_{3}\right)}$$

$$B=i_{4}⊕\bar{\left(i_{7}\vee i_{6}\right)}$$

$$C=i_{6}⊕\bar{\left(i_{1}\vee i_{2}\right)}$$

$$D=i_{5}⊕\bar{\left(A\vee B\right)}$$

$$E=i_{1}⊕\bar{\left(A\vee i_{3}\right)}$$

$$F=i_{7}⊕\bar{\left(C\vee D\right)}$$

$$G=i_{3}⊕\bar{\left(B\vee D\right)}$$

$$H=i_{2}⊕\bar{\left(E\vee F\right)}$$

Finally, the output of *SubCell* is as shown,

$y_{p}=s_{7}∥s_{6}∥s_{5}∥s_{4}∥s_{3}∥s_{2}∥s_{1}∥s_{0}⇐D∥B∥A∥G∥E∥C∥F∥H$ where *p* = 0,1,2,‥15.

During the *Addconstants* operation, the constant values denoted as *ac*_0_, *ac*_1_, and *ac*_2_ in the array ‘*A*_*c*_‘ are added to the respective cell position of the cipher state.


$$A_{c}=[\begin{array}{cccc} ac_{0} & 0 & 0 & 0\\ ac_{1} & 0 & 0 & 0\\ ac_{2} & 0 & 0 & 0\\ 0 & 0 & 0 & 0 \end{array}]$$

A 6-bit linear feedback shift register (LFSR) is deployed to update the round constant state. The state of LFSR is expressed as (*rc*_5_,*rc*_4_,*rc*_3_,*rc*_2_,*rc*_1_,*rc*_0_). The initial state bits of LFSR hold zero value which are updated before every next encryption round. The constant values are updated for each encryption round. The update function is described as $\left(rc_{5}∥rc_{4}∥rc_{3}∥rc_{2}∥rc_{1}∥rc_{0})\to (rc_{4}∥rc_{3}∥rc_{2}∥rc_{1}∥rc_{0}∥rc_{5}⊕rc_{4}⊕1\right)$. The value of *ac*_0_, *ac*_1_ and *ac*_2_ are as expressed as below,

$$ac_{2}=0\times 2;\;(ac_{0},ac_{1})=(0∥0∥0∥0∥rc_{3}∥rc_{2}∥rc_{1}∥rc_{0},0∥0∥0∥0∥0∥0∥rc_{5}∥rc_{4})$$

**Fig 4:**
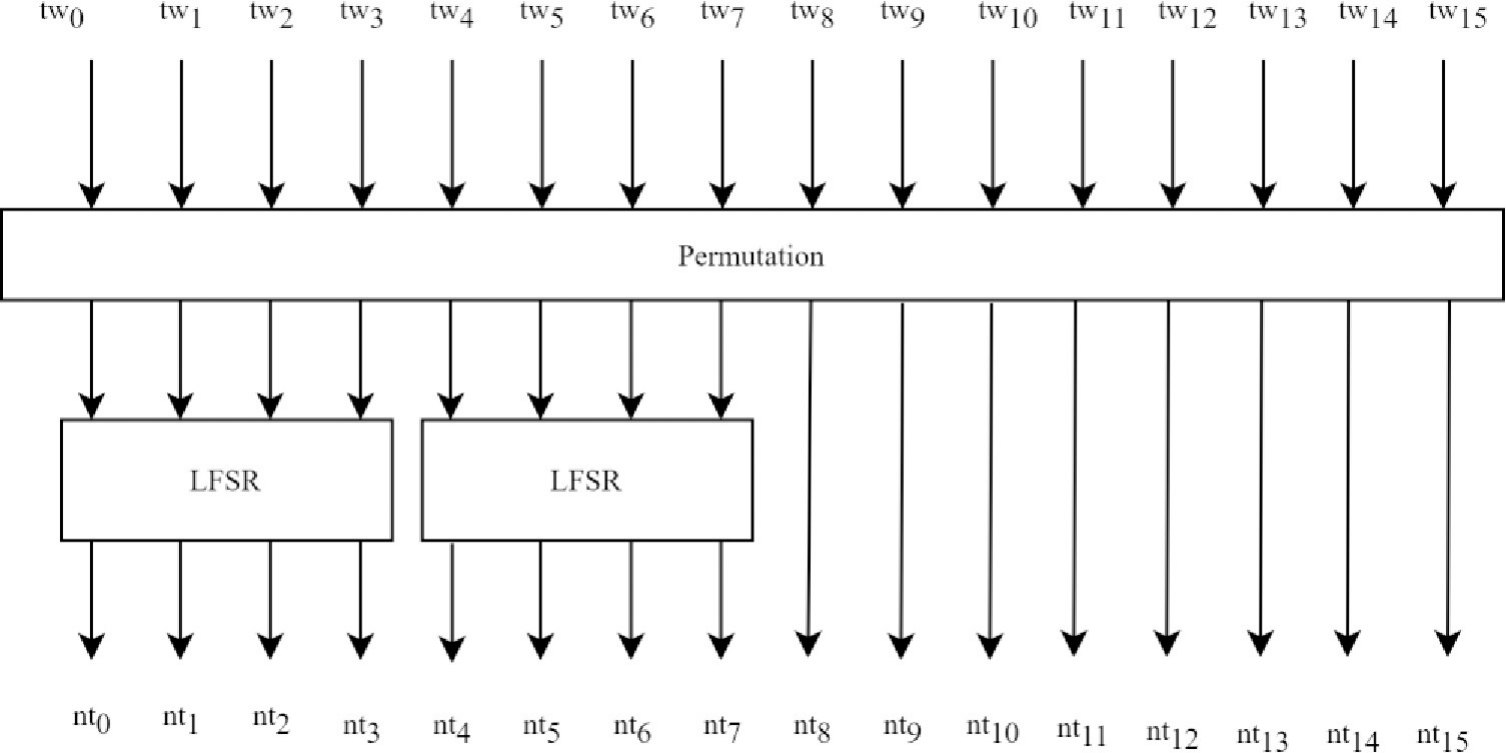
Tweakey updating schedule.

In the *AddRoundTweakey* operation, the first two rows of the tweakey array are ex-ored with the respective cell position of the cipher state. The tweakey arrays are updated in each round as shown in Fig 4.

A permutation is applied to the cell positions of all tweakey arrays, as shown below.


$$\left(tw_{0},....,tw_{15})\to (tw_{9},tw_{15},tw_{8},tw_{13},tw_{10},tw_{14},tw_{12},tw_{11},tw_{0},tw_{1},tw_{2},tw_{3},tw_{4},tw_{5},tw_{6},tw_{7}\right)$$

After permutation, each cell of the first and second rows of tk2 and tk3 are updated with an LFSR. If the primary member is in use, both tk2 and tk3 arrays are updated; for the secondary member, only the tk2 array is updated. Each tweakey is updated with a similar transformation schedule. For tk2, LFSR updates 8-bits of each cell as below,

$$\left(a_{7}∥a_{6}∥a_{5}∥a_{4}∥a_{3}∥a_{2}∥a_{1}∥a_{0})\to (a_{6}∥a_{5}∥a_{4}∥a_{3}∥a_{2}∥a_{1}∥a_{0}∥a_{7}⊕a_{5}\right)$$

For tk3, LFSR updates 8-bits of each cell as below,

$$\left(a_{7}∥a_{6}∥a_{5}∥a_{4}∥a_{3}∥a_{2}∥a_{1}∥a_{0})\to (a_{0}⊕a_{6}∥a_{7}∥a_{6}∥a_{5}∥a_{4}∥a_{3}∥a_{2}∥a_{1}\right)$$

In the *ShiftRows* operation, the cell rows of the state except the first row are rotated to the right by 1, 2, and 3 positions, respectively. Finally, during the *MixColumns* operation, columns of the state matrix are multiplied by a binary matrix ‘*M*‘.


$$M=(\begin{array}{cccc} 1 & 0 & 1 & 1\\ 1 & 0 & 0 & 0\\ 0 & 1 & 1 & 0\\ 1 & 0 & 1 & 0 \end{array})$$

The *MixColumn*, in combination with the *ShiftRows* operation, are performed to add another layer of diffusion. To offer the best security/performance tradeoff, the binary matrix *M* with a branching number of two is used. Multiplication with “0” and “1” does not require any processing. Multiplication followed by exclusive-OR operation is performed during the *MixColumn* operation. The output array of the *MixColumn* operation is called ciphertext. A detailed description of SKINNY-Hash is presented in [31].

## Hybrid redundancy

**Fig 5:**
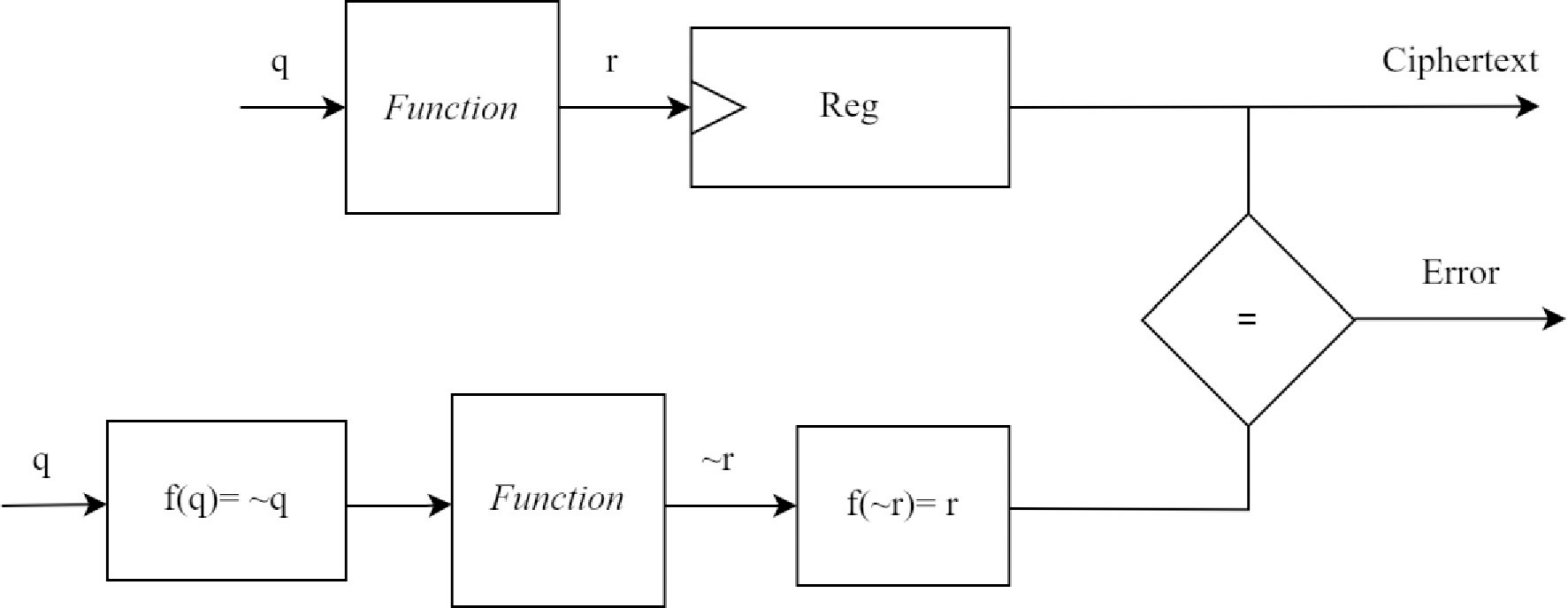
Hybrid redundancy.

This section elaborates on the hybrid redundancy used for fault detection in the lightweight SKINNY block cipher. Time and hardware redundancy offers high reliability against transient faults. However, a permanent fault can not be detected if it exists in redundant copies. Time or hardware redundancy combined with an encoding scheme can detect permanent and transient faults, improving fault detection coverage. Redundancy can be constructed in such way that the computation does not affect the desired output. Alternating logic is a simple and efficient encoding method to detect permanent faults. The self-dual function for input assignment *x* equals the value of the complement of function for input assignment ~*x* [32]. In this work, the self-dual function is used to get complemented operands for recomputation and further to get complemented output for comparison, as shown in Table 1. In self-dual function, $f(p,q,r)=\bar{f}(\bar{p},\bar{q},\bar{r})$ is true. Fig 5 shows hybrid redundancy where the self-dual function is used during recomputation for fault detection.

**Table 1:** Truth table for self-dual function.

(*p*,*q*,*r*)	*f*(*p*,*q*,*r*)	$f(\bar{p},\bar{q},\bar{r})$	$\bar{f}(\bar{p},\bar{q},\bar{r})$
000	***f***_**0**_	***f***_**7**_	$\bar{f_{7}}$
001	***f***_**1**_	***f***_**6**_	$\bar{f_{6}}$
010	***f***_**2**_	***f***_**5**_	$\bar{f_{5}}$
011	***f***_**3**_	***f***_**4**_	$\bar{f_{4}}$
100	***f***_**4**_	***f***_**3**_	$\bar{f_{3}}$
101	***f***_**5**_	***f***_**2**_	$\bar{f_{2}}$
110	***f***_**6**_	***f***_**1**_	$\bar{f_{1}}$
111	***f***_**7**_	***f***_**0**_	$\bar{f_{0}}$

### Application of hybrid redundancy to SKINNY

**Fig 6:**
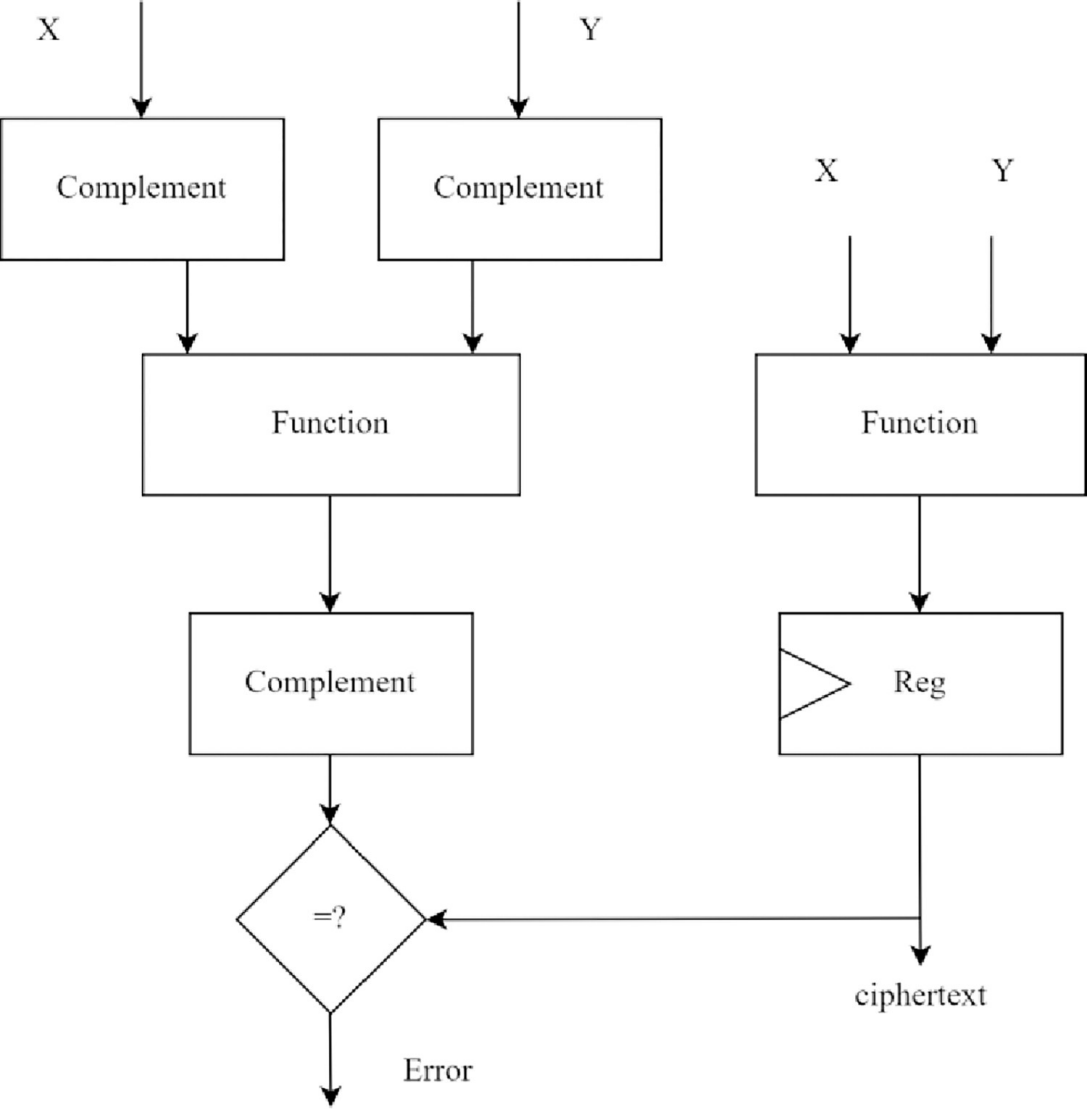
Hybrid redundancy for SKINNY block cipher.

In this section, hybrid redundancy for the SKINNY-block cipher is elaborated. Fig 6 shows RECO architecture, where the function *f* is the SKINNY encryption round, and the self-dual boolean function is the complement operation.

Two SKINNY encryption computation rounds are performed; each round performs five operations. The first computation round *R*_1_ encryption is performed with normal operands, and the second computation round *R*_2_ encryption is performed with complemented operands. The *SubCell*, *AddConstant*, and *AddRoundTweakey*are implemented using combinational logic. Any single-bit fault injected during *SubCell*, *AddConstant*, and *AddRoundTweakey* is reflected in gate-level implementation. The *ShiftRows* operation implemented in ASIC or FPGA is only a wire connection. Hence, any single-byte or single-bit fault injected during the *ShiftRows* operation will be an input to *MixColoumns*. The *MixColoumns* is implemented using exclusive-OR, and the injected fault will propagate to other cells of the state vector. It is observed that the output of each operation in *R*_2_ is complement of *R*_1_ round outputs except for *MixColumns* operation. The ciphertext generated after one round of SKINNY block cipher is shown below,

Output of *R*_1_ *MixColumns* operation is

$$ciphertext=[\begin{array}{cccc} c_{0} & c_{1} & c_{2} & c_{3}\\ c_{4} & c_{5} & c_{6} & c_{7}\\ c_{8} & c_{9} & c_{10} & c_{11}\\ c_{12} & c_{13} & c_{14} & c_{15} \end{array}]$$

Output of *R*_2_ *MixColumns* operation is

$$\bar{ciphertext}=[\begin{array}{cccc} \bar{c_{0}} & \bar{c_{1}} & \bar{c_{2}} & \bar{c_{3}}\\ \bar{c_{4}} & \bar{c_{5}} & \bar{c_{6}} & \bar{c_{7}}\\ c_{8} & c_{9} & c_{10} & c_{11}\\ c_{12} & c_{13} & c_{14} & c_{15} \end{array}]$$

The cipher text generated after *R*_2_ computation differs from *R*_1_ computation only in the first two rows. The exclusive-OR operation involved in *MixColumns* causes the $\bar{ciphertext}$ to have each cell in the last two rows similar to the respective cell in *ciphertext*. The first row’s cell values are computed with two exclusive-OR, and the second row is obtained by a shift-row operation; hence, the first two rows are the complement of its respective cell values in the *ciphertext*. These two rows of $\bar{ciphertext}$ need to be complemented before comparing the outputs of *R*_1_ and *R*_2_ rounds of computation. The third and fourth rows do not need to complement, reducing hardware overhead. After complementing the first two rows and comparing the two ciphertexts, a fault is detected if the output does not match. One round of SKINNY-Hash function is expressed as shown,

$SKINNY_{r}(ciphertext)=(MxC(ShR(AdT(AdC(SbC(Q))))))$; where *Q* is the internal state vector, and *r* is the total number of rounds depending on block size and tweaked size.

### Proposed architecture implementation and simulation results

Redundancy is implemented at two levels: low-level and high-level. Low-level redundancy offers higher reliability than high-level redundancy. Hence, low-level redundancy for the SKINNY cipher is chosen to improve the dependability of the SKINNY-Hash function. Iterative architecture is adopted for the SKINNY block cipher. In iterative architecture, the ciphertext is produced after 48 rounds in the primary hash function and after 56 rounds in the secondary hash function. To enhance security, one can check the output of each round or after several rounds based on the tradeoff between reliability, security, and performance. The faulty ciphertext can be prevented from being generated if faults are detected before it is produced.

#### Recomputing with complemented operands

**Fig 7:**
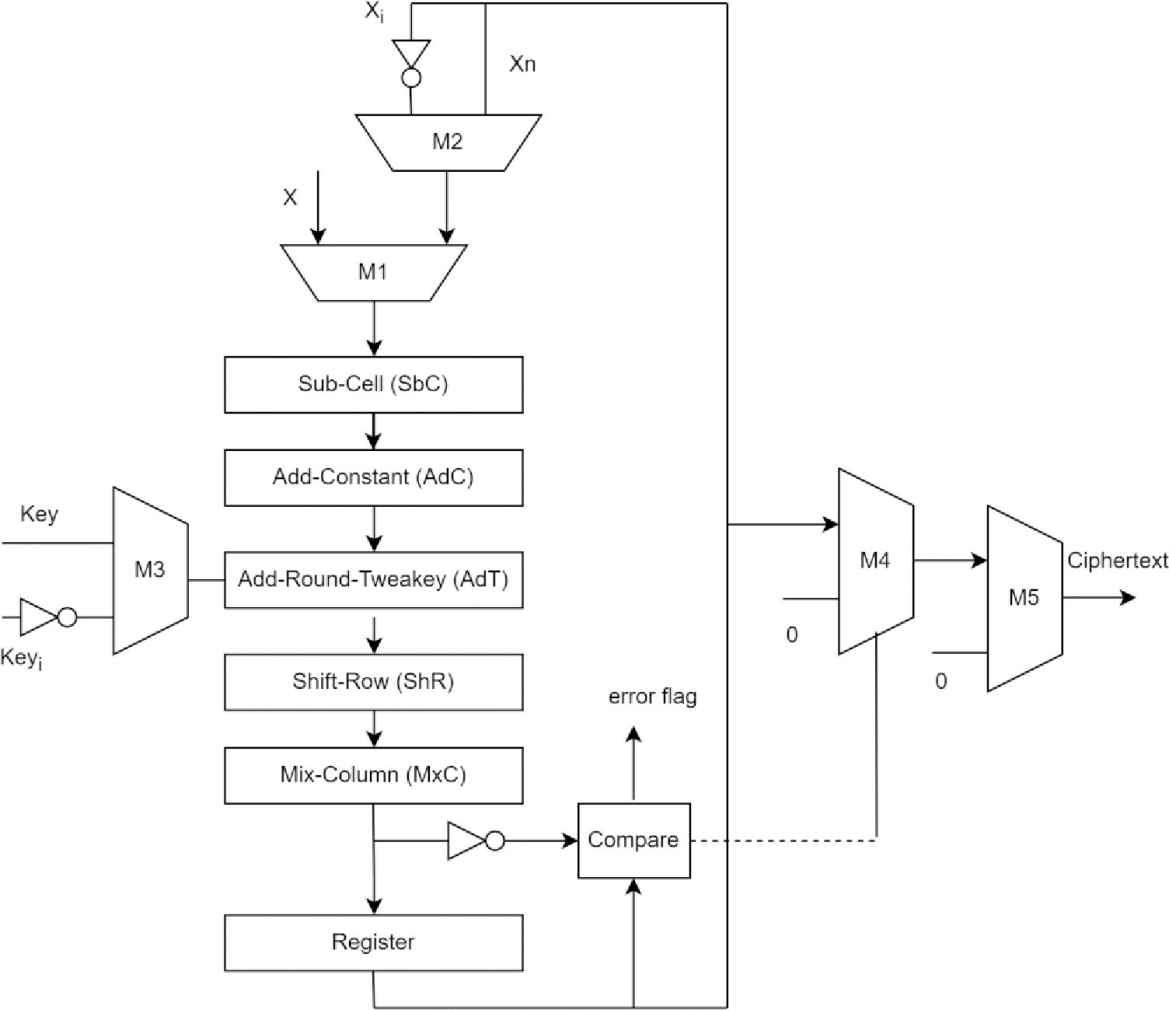
Implementation of SKINNY-Hash function, including RECO.

In this section, RECO for the SKINNY-block cipher is presented, as shown in Fig 7. The encryption round in the function block is computed twice. The first encryption cycle *C*_1_ takes normal input operands, i.e. *X* as plaintext and *Key*. The inputs are chosen using *mux M*_1_ and *muxM*_3_. The second encryption cycle *C*_2_ takes complemented operands, i.e. *X*_*i*_ and *Key*_*i*_. The inputs are chosen using *mux M*_2_ and *muxM*_3_. *C*_1_ produces output, which is stored in the register. The output is then fed to the *muxM*_2_ for *C*_2_ cycle where it is complemented and *X*_*i*_ is fed for recomputation. The *C*_2_ output obtained is complemented before being compared it with the *C*_1_ output previously saved in the register. The compare operation sets the error flag signal if the outputs of *C*_1_ and *C*_2_ do not match. For the next encryption rounds, the updated *Key* and *X*_*n*_ are given as input. The error flag is fed to output *muxM*_4_ as a select signal. The error flag is set once a fault is detected, and the *muxM*_4_ output is produced as “0”. Otherwise, the stored register value is produced by *mux M*_4_. The control logic is developed to initiate the encryption in the architecture, to set/reset select signal for mux, and to produce round constants. Once all the rounds are executed, the select signal for *mux M*_5_ is initiated to generate the final ciphertext. Otherwise, once the error signal is set, the control unit initiates the select signal to *mux M*_5_ and produces “0” as ciphertext.

#### Double modular redundancy with complemented operands

Hardware redundancy improves the reliability of digital systems at the expense of more area-power overhead.

**Fig 8:**
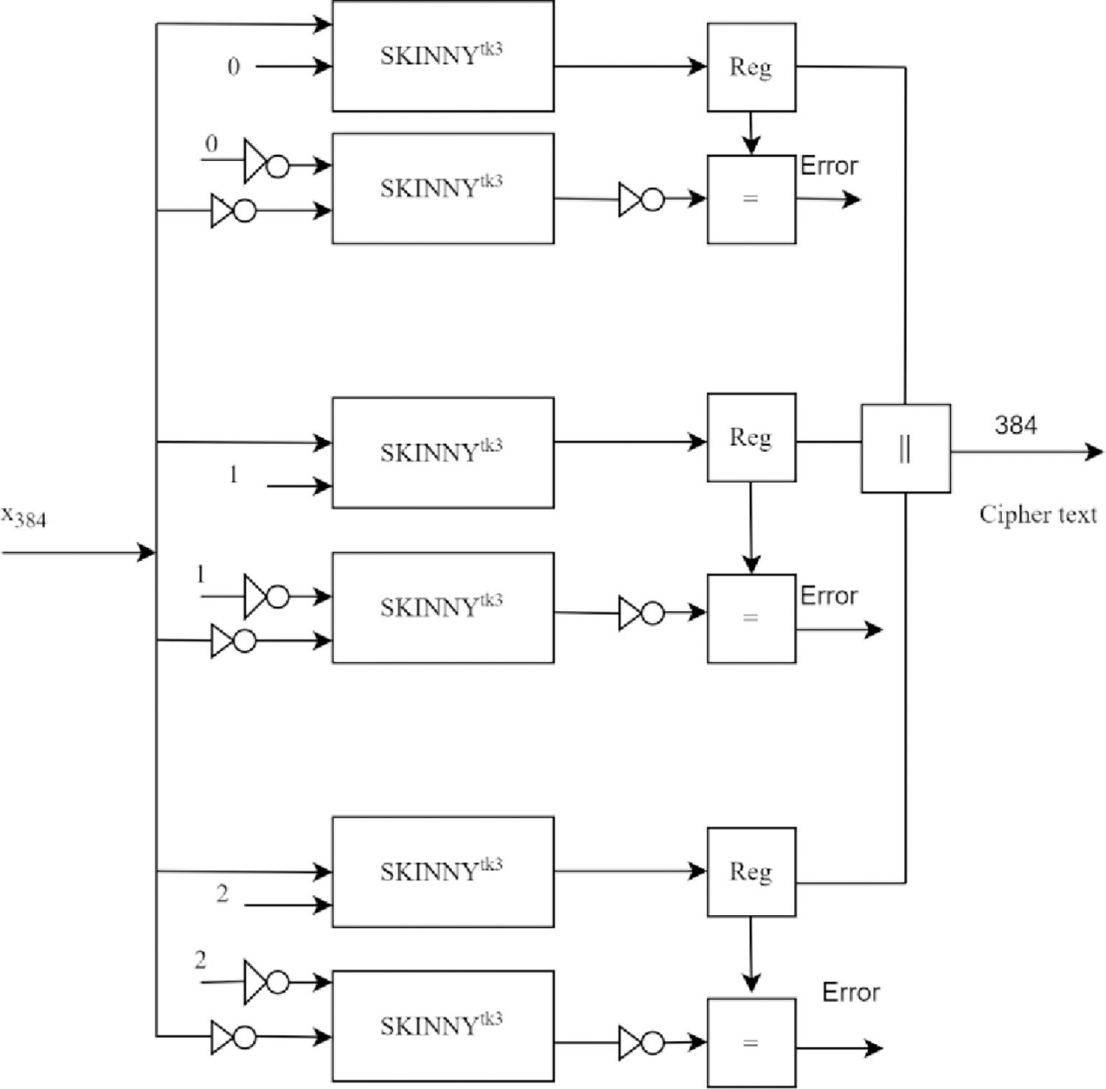
Implementation of SKINNY Hash function, including DMR.

Triple modular redundancy (TMR) is a popular fault detection technique. Three redundant copies of the hardware under test are created, and the output is chosen after voting. To achieve low power consumption, DMR is proposed in [33]. Two redundant copies are created, and the output is compared to detect faults in the hardware. SKINNY block cipher with DMR is shown in Fig 8.

**Fig 9:**
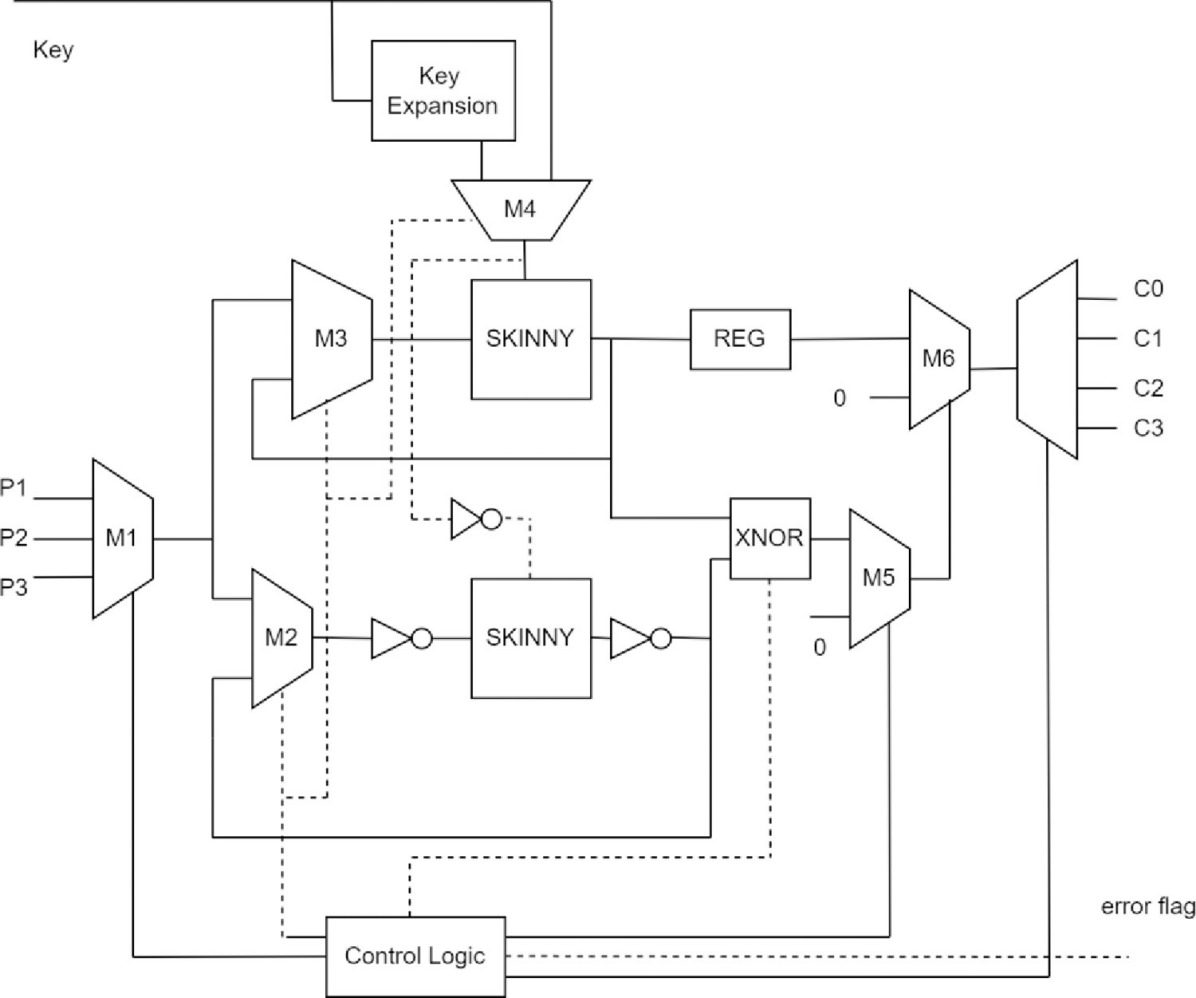
Implementation of SKINNY Hash function, including DMRC.

For architectural improvement in terms of low-area and low-power consumption, resource sharing is proposed for the hash function, as shown in Fig 9. In complex digital circuits, one module is shared via multiplexers inserted at its inputs and outputs to enable resource sharing across multiple operations in the design to minimise hardware overhead [34, 35]. Hence, the SKINNY block cipher is shared in the hash function to optimise area and power. Here, a control logic for the Hash function is implemented to share the SKINNY block promptly for ciphertext computation.

The complemented plaintext and tweakey are given as input to the redundant copy of the SKINNY block cipher. Unlike time redundancy, each encryption round is performed once on the respective redundant copy. To lessen area and power overhead, the DMRC model of SKINNY is shared in a timely manner within the hash function. The control logic is developed to share the SKINNY block cipher. The control logic initiates the encryption and takes in input plaintext and key using *mux M*_1_, *M*_2_, *M*_3_, and *M*_4_.The SKINNY block ciphers encrypt the plaintext and generate output. The outputs of redundant copies are compared using exclusive OR. The comparison output is forwarded to the control logic. If the comparison output is not “1”, then the fault is detected. The control logic sets an error flag, initiates select signals of *muxM*_5_, and *M*_6_ once the fault is detected. The final ciphertext is forced to produce “0” using mux *M*_5_, and *M*_6_. If no fault is detected, the next round of encryption takes place with the updated key and ciphertext produced by the previous round. The final ciphertext *C*_1_ is generated after 56 rounds of SKINNY block cipher in *SKINNY*^*ik*3^. Further, *P*_2_ is taken as the next plaintext for encryption and *C*_2_ is produced after 56 rounds of encryption. If no fault is detected, then the encryption continues to produce ciphertext *C*_3_. Each ciphertext is 128-bit and is stored in the 384-bit register.

#### Area and power analysis of RECO and DMRC

According to the authors’ knowledge, there is no work in previous literature that proposed low area and low power fault detection techniques for SKINNY-Hash. Hence, other fault detection techniques proposed in the literature are applied to SKINNY-tk2 and SKINNY-tk3 hash functions for comparison regarding area and power consumption. The power consumption in any architecture majorly depends on the leakage current and switching activity of logic states during the operations [36]. In RECO and DMRC, NOT gates are used to complement the input bits for recomputation, and the complement operation consumes less power than any other encoding scheme in the literature. The RECO architecture follows the traditional time redundancy technique; hence, the recomputation causes more power consumption than the actual SKINNY-Hash function architecture. Therefore, DMRC is proposed where less power consumption can be achieved with early fault detection. Let the total number of cycles for a normal SKINNY-Hash function be “*n*_*c*_” cycles; then RECO will take “2*n*_*c*_” cycles. DMRC will take “3*n*_*c*_” cycles for primary member and “2*n*_*c*_” cycles for secondary member.

The results are tabulated in Table 2, showing the area and power consumption of the SKINNY-Hash with no CED and with CED in function blocks. SKINNY-Hash is synthesized to generate area-power reports using the Genus tool by Cadence using 90nm CMOS process technology, where the supply voltage is 0.9 volt and frequency is 2.8GHz. The percentage values are calculated with respect to the SKINNY-Hash function block.

**Table 2:** Implementation results of SKINNY-Hash algorithm with 128-bit block size.

	Tweakey size	Area (mm^2^)	Total power (mW)
**SKINNY-Hash**	256	0.6749	94.924
**SKINNY-Hash with REPO [22]**	256	2.2067(69.4%)	143.029(33.6%)
**SKINNY-Hash with CED [23]**	256	1.26(46.6%)	137.654(31.04%)
**SKINNY-Hash with DMR [33]**	256	1.7808(62%)	129.767(26.8%)
**SKINNY-Hash with RECO (Proposed work)**	256	0.9201(26.6%)	124.299(23.6%)
**SKINNY-Hash with DMRC (Proposed work)**	256	0.6988(3.4%)	102.997(7.84%)
**SKINNY-Hash**	384	0.6536	79.017
**SKINNY-Hash with REPO [22]**	384	6.1801(89.4%)	621.019(87.3%)
**SKINNY-Hash with CED [23]**	384	2.564(74.5%)	584.254(86.4%)
**SKINNY-Hash with DMR [33]**	384	3.9894(83.6%)	538.256(85.3%)
**SKINNY-Hash with RECO (Proposed work)**	384	2.0479(68%)	254.658(68.9%)
**SKINNY-Hash with DMRC (Proposed work)**	384	0.6988(3.4%)	178.953(55.8%)

The proposed work uses a self-dual Boolean function with time and hardware redundancy; it adds low hardware overhead compared to previous work proposed in the literature. The number of internal rounds depends on the tweakey size ‘t’. For block size n = 128 and tweakey t = 2n, the area overhead is 69.4%, 62%, 26.64%, and 3.42% for REPO, CED, DMR, RECO, and DMRC, respectively. For block size n = 128 and tweakey t = 3n, the area overhead is 89.4%,74.5%, 83.6%, 68.08%, and 50.4% for REPO, CED, DMR, RECO, and DMRC, respectively. For block size n = 128 and tweakey t = 2n, the power overhead is 33.63%, 31.04%, 26.8%, 23.63%, and 7.84% for REPO, CED, DMR, RECO, and DMRC, respectively. For block size n = 128 and tweakey t = 3n, the power overhead is 87.27%, 86.4%, 85.3%, 68.97%, and 55.84% for REPO, CED, DMR, RECO, and DMRC, respectively. It is observed that with DMRC, the area and power consumption are reduced compared to RECO and other techniques.

#### Fault analysis of RECO and DMRC

The behavioural functionality of the proposed work is observed using Mentor Graphics ModelSim SE-64. Fault simulations for RECO and DMRC are performed to verify the functionality of proposed architectures. To assess fault coverage, fault bits were injected at random locations, such as inputs and outputs of each internal round operation and detected faults were monitored. Fault coverage is a measure of how accurately the test vectors detect faults in digital design. It is a ratio of the number of detected faults to the total detectable faults. The fault coverage is calculated as shown,

$$\mathrm{Fault}\;\mathrm{coverage}=\frac{\mathrm{Detected}\;\mathrm{faults}}{\mathrm{Total}\;\mathrm{detectable}\;\mathrm{faults}}\times 100$$

As for single-bit, the faulty bits are injected into the input and output logic lines. The output lines of one operation act as input for the next operation in the encryption round. The architecture takes 128-bit input and includes 5 operations in each encryption round, hence 128×5×2 = 1280 single faulty bits were injected into the design. The *SubCell* operation is implemented using exclusive-OR and NOR gate. Hence, any fault injected at the input lines of S-box will reflect in the output of S-box. The *AddConstant* and *AddRoundTweakey* operation are implemented using combinational logic circuits. Therefore, any faults injected at the input of these operation reflects in gate level implementation. The ShiftRows operation is the wired connection between the output of *AddRoundTweakey* and input of *MixColumns* operation. Again, MixColumns operation is implemented using exclusive-OR and the faulty input bits causes faulty ciphertext generation.

A linear feedback shift register (LFSR) is implemented to inject faults through the testbench for multiple-bit and single-byte fault injection. An 8-bit registers are used to inject faults in each cell of the internal state. A 2:1 multiplexer is used to inject either a faulty bit or an actual input bit. These multiplexers are placed at locations where faults are to be injected. The select signal of these multiplexers is handled by another LFSR to inject faulty bits randomly. The LFSR registers produce a pseudorandom sequence which flips random bits in the output bit at random intervals. The feedback polynomial for the 8-bit registers is *x*^8^+*x*^6^+*x*^5^+*x*^4^+1. Through LFSR, around 9×10^9^ faults were injected in total at the operation inputs. Single-bit, single-byte, and multiple-bit faults were detected, and the achieved fault coverage is shown in Table 3. The single-bit, single-byte and multiple bit faults are injected at random instances as transient faults, and the achieved fault coverage is very high. The fault bits are not detected in cases where the injected fault bit is similar to the actual input bit.

**Table 3:** Fault detection capability of RECO and DMRC.

Fault types	Fault coverage (%)		
	Single bit	Single byte	Multiple bits
**REPO [22]**	100%	100%	99.9999997%
**DMR [33]**	100%	100%	100%
**CED [23]**	88.58%	98.8%	99.856%
**RECO**	100%	99.991%	99.996%
**DMRC**	100%	100%	99.991%

For permanent fault detection, the input bits and output bits are forced either to be stuck at ‘0’ or ‘1’. The achieved fault coverage for permanent faults is 100%. Comparing state-of-the-art fault-detection techniques for the SKINNY algorithm in terms of area and total power consumption proves that the proposed work provides low area-power overhead.

## Conclusion

In this paper, two approaches for low-power fault detection are proposed for the SKINNY-Hash function. Two hybrid redundancy techniques are proposed: time redundancy with an encoding scheme (RECO) and hardware redundancy with an encoding scheme (DMRC). These techniques are applied to primary and secondary members of the hash function in the lightweight SKINNY-Hash algorithm to compute a secure hash digest. The simulation results prove that the proposed work has approximately 100% fault coverage. The RTL is written in VHDL and synthesised using 90nm CMOS process technology. The present fault detection techniques available in the literature are also applied to SKINNY-Hash functionality. The CMOS implementation outcome shows a notable reduction in power consumption compared to other fault detection techniques with less overhead in the area. For future work, architecture level low power design techniques can be implemented to reduce further the power consumption caused by redundancy. The control unit in the architecture also needs to be considered for fault detection.
